# Incidence, Clinical Characteristics, and Genotype Distribution of Rotavirus in a Neonatal Intensive Care Unit 5 Years After Introducing Rotavirus Vaccine

**DOI:** 10.3389/fped.2022.850839

**Published:** 2022-02-17

**Authors:** Hye Sun Yoon, Jiseun Lim, Yong-Hak Sohn, Seung Yeon Kim

**Affiliations:** ^1^Department of Pediatrics, Nowon Eulji Medical Center, Eulji University School of Medicine, Seoul, South Korea; ^2^Department of Preventive Medicine, Eulji University School of Medicine, Daejeon, South Korea; ^3^Seegene Medical Foundation, Seoul, South Korea; ^4^Uijeongbu Eulji Medical Center, Eulji University School of Medicine, Uijeongbu, South Korea

**Keywords:** rotavirus, newborn, vaccination, genotype, neonatal intensive care unit (NICU)

## Abstract

**Background:**

Rotavirus (RV) is a common cause of viral gastroenteritis in children worldwide. We aimed to investigate the incidence, symptoms, and genotype of RV infection in a neonatal intensive care unit (NICU) in South Korea 5 years after the introduction of RV vaccination to evaluate its effect on newborn infants.

**Methods:**

A total of 431 fecal specimens were collected from patients admitted to NICU between April 20, 2012 and September 10, 2013. Enzyme-linked immunoassays were used to detect RV antigen. Nested multiplex polymerase chain reaction was used for genotyping.

**Results:**

The overall incidence of RV infection was 43.9% and was significantly higher in preterm infants, infants born in the study hospital, low birth weight infants, and cesarean births (*P* < 0.05). Symptoms of diarrhea, poor feeding, abdominal distension, and apnea were significantly higher in infants with RV infection than those without infection. RV infection gradually increased depending on infant care at home, postpartum clinic, or hospital (26.0, 45.1, and 60.2%, respectively; *P* = 0.000). The dominant RV genotype in the NICU was G4P[6] at 95.4%.

**Conclusion:**

Current RV vaccines did not affect the incidence of RV infection in newborn and preterm infants in the NICU. Most RV-positive patients in the NICU had symptoms, and the incidence of RV infection was relatively higher in hospitals and postpartum clinics with group life than home. The dominant RV genotype was G4P[6] across study groups.

## Introduction

Rotavirus (RV) is the predominant cause of acute viral gastroenteritis in infants and young children. In 2016, diarrheal illness from RV infection caused 128,500 deaths (95% uncertainty interval [UI], 104,500–155,600) among children younger than 5 years; thus, 28.8% (95% [UI], 25.0–32.6%) of the deaths from diarrhea in this age group were reported to be caused by RV (1). The overall burden of RV infection has been significantly reduced by the approval of a number of RV vaccines for use in routine childhood immunization series. However, it continues to cause illness in infants, especially in developing countries where RV vaccines are not readily available (2, 3). In addition, RV infection can affect newborn infants who are too young to receive vaccination. Newborn infants in hospitals can be infected with RV at the nursery and neonatal intensive care units (NICU), and symptoms can be mild or asymptomatic (4–9). However, preterm infants are at a greater risk of severe RV infection. Previous studies have shown that preterm infants have higher hospitalization rates and more severe gastrointestinal symptoms, including intestinal dilatation, abdominal distension, and mucoid stools, than term infants (9).

Two vaccines, RotaTeq (RV5, Sanofi Pasteur MSD, Lyon, France) and Rotarix (RV1, GSK Biologicals, Rixansart, Belgium), were launched in 2007 in South Korea to prevent RV infection. After vaccination in the general population, the prevalence of RV infection was reduced in South Korea, but there was an increase in RV infection among newborns and children under 2 months of age, who were ineligible for the RV vaccines (10). This outcome is not unique to South Korea; therefore, many countries are trying to develop an effective RV vaccine for newborn infants (11). Information of genotype and incidence of RV is needed to succeed immunization of RV in newborn as well as preterm infants because RV incidence and genotype are changeable as time and region (12–14). This study aimed to investigate the incidence, symptoms, and genotype of RV infection in NICU after introducing RV vaccination in a single medical center in South Korea.

## Materials and Methods

### Research Subjects and Materials

Between April 20, 2012 and September 10, 2013, 431 fecal samples from admitted newborn infants were collected in the NICU at Eulji University Hospital, South Korea. Newborn infants before 28 days of age were admitted to our NICU, and the mean age of the infants birthed outside of the hospital (outborn group) was 8.6 days. All outborn infants were tested for RV infection upon admission to the hospital. The outborn group and infants birthed in the hospital (inborn group) received a RV antigen test every 2 weeks, even if they were asymptomatic, to check for nosocomial infection. Further, infants were tested if there were any of the following symptoms during hospitalization: (1) Loss of appetite with vomiting and watery diarrhea, (2) fever or apnea with no apparent symptoms or signs of respiratory or systemic bacterial infection, and (3) feeding intolerance with abdominal distension and residue. Even if an infant was tested multiple times, we classified the infant as RV-positive if a positive result was found on screening or symptomatic tests, and RV-negative otherwise. The clinical information of the infants was collected by a questionnaire completed by each of their parents.

### Research Method

#### RV Antigen Detection

Detection of RV antigen was performed using VIDAS Rotavirus (bioMerieux Vitek, France) or RIDASCREEN Rotavirus (R-Biopharm AG, Germany) immunoassay. The detection of antigen by both test methods showed the comparable results (15).

#### Preservation of Specimen and Pre-analysis of Genotype

Fecal samples for RV genotyping were stored in a −70°C freezer, and thawed at room temperature before analysis. Then, 0.5–1.0 × 10^−3^ kg of feces was dissolved in 5 mL of 0.89% normal saline and centrifuged at 4,000 g. After centrifugation, 140 μL of supernatant was used for genotyping.

#### RV Genotype Analysis

RV genotyping was performed using multiplex polymerase chain reaction (PCR), and sequencing was performed when it was difficult to analyze the genotypes by multiplex PCR. G and P genotypes along with two outer capsid proteins, VP7 (glycoprotein) and VP4 (protease sensitive protein), were analyzed using reverse transcription-PCR (RT-PCR) after extracting RNA from fecal samples using the QIAamp viral RNA mini kit (Qiagen GmbH, Hilden, Germany) (16, 17). RT-PCR was performed using Beg9 and End9 primers to determine G genotypes. Nested multiplex PCR was also performed using End9 and type-specific primers (aBT, aCT2, aFT3, aDT4, aAT8, and aFT9) to determine G genotypes. RT-PCR was performed using Con2 and Con3 primers and nested multiplex PCR was performed using Con3 and type-specific primers (1-T1, 2-T1, 3-T1, and 4-T1) to determine P genotypes. PCR reaction conditions followed the protocols described in published papers (16, 17). Ten μL of each PCR product was electrophoresed on 1.5% agarose gel (Sigma-Aldrich, St. Louis, MO, USA), stained with ethidium bromide, and observed under ultraviolet illumination. When the genotypes were not determined by nested multiplex PCR after amplification in primary RT-PCR, sequencing analysis was performed using primary primers for primary RT-PCR products. Genotype was determined by phylogenetic analysis using the Claustral Omega program (http://www.ebi.ac.uk/Tools/msa/clustalo/) of the European Bioinformatics Institute. RV reference strains for phylogenetic analysis were selected by referring to a study by Matthijnssens (18).

### Statistical Analysis

All results were analyzed using SPSS 20.0 (IBM, Chicago, IL, USA). Pearson's chi-square test and Chi-square test for trend analysis were used. *P* < 0.05 was considered statistically significant.

The study protocol was reviewed and approved by the Institutional Review Board of Eulji University Hospital (12-064). Informed consent was obtained from all parents of the subjects on enrollment.

## Results

### Incidence of RV

**Figure 1:**
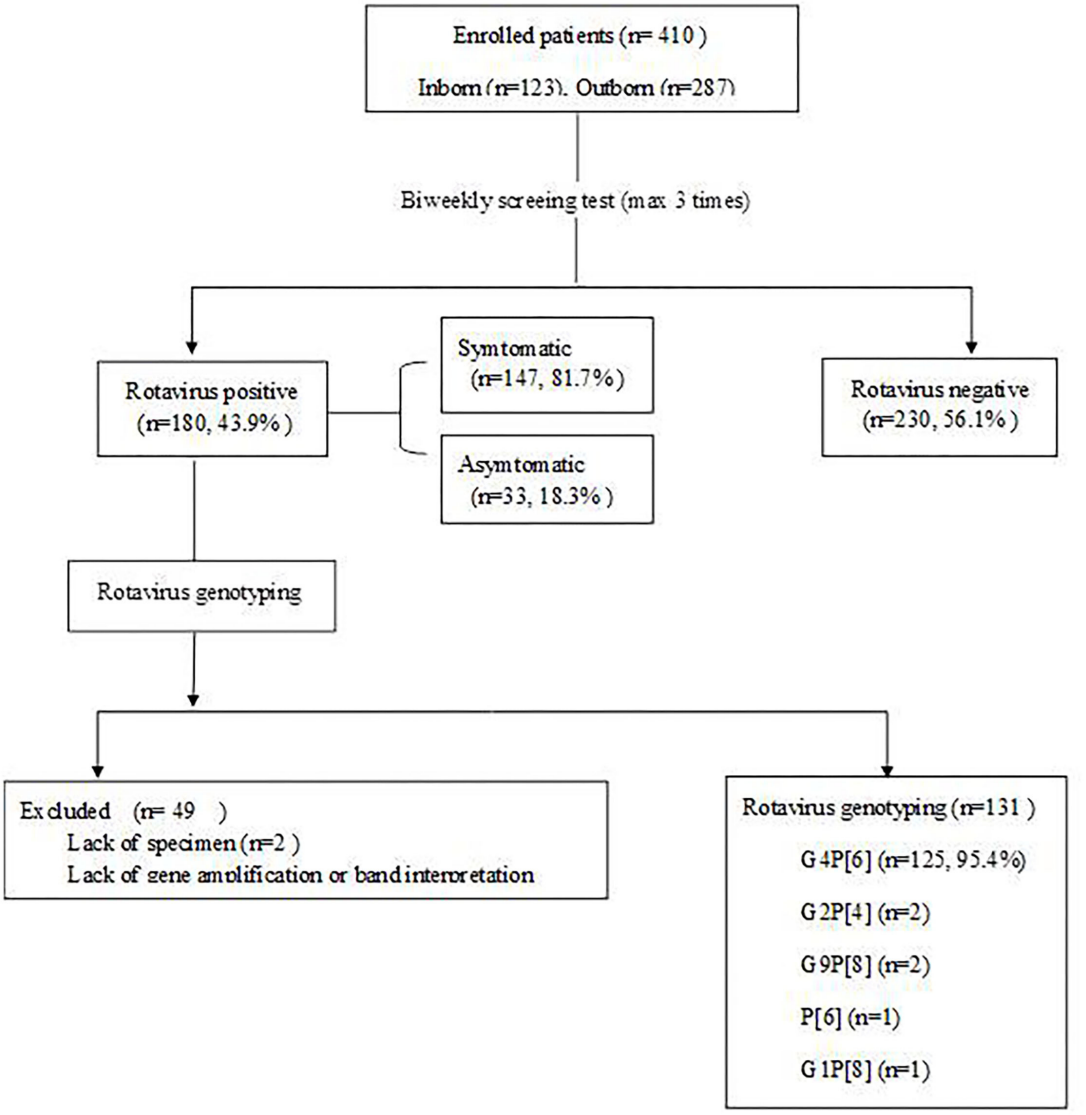
Flow diagram of study population.

RV antigens were tested in 410 of 431 fecal samples of patients whose parents provided consent for participation in the study; 21 samples were missing. A total of 180 specimens (43.9%) were positive by the RV antigen test (Figure 1). Statistically significant difference was not observed with respect to the gender of the patients. Patients with a birth weight of ≤ 2,500 g had a significantly higher incidence of RV infection than patients with a birth weight >2,500 g (*P* = 0.000). Incidence of RV infection was significantly higher in the group of infants younger than 37 gestational weeks than those older than 37 gestational weeks (*P* = 0.000). The cesarean section group had a significantly higher incidence of RV infection than the vaginal delivery group (*P* = 0.001). The inborn group had a statistically significantly higher incidence of RV infection than the outborn group (*P* = 0.000; Table 1).

**Table 1:** Demographic characteristics of rotavirus positive cases and negative cases in NICU.

	**Number of patients (%)**		
**Demographics**	**RV (+)**	**RV (–)**	***P*-value**
**Gender**			
Male	97 (41.8)	135 (58.2)	0.318
Female	83 (46.6)	95 (53.4)	
**Birth weight**			
≦2,500 g	57 (60.0)	38 (40.0)	0.000^*^
>2,500 g	123 (39.0)	192 (61.0)	
**Gestational age**			
<37 weeks	63 (61.8)	39 (38.2)	0.000^*^
≧37 weeks	116 (37.7)	192 (62.3)	
**Labor**			
Normal delivery	92 (36.9)	157 (63.1)	0.001^*^
Cesarean section	87 (54.0)	74 (46.0)	
**Birth place**			
Inborn infant	80 (65.0)	43 (35.0)	0.000^*^
Outborn infant	100 (34.8)	187 (65.2)	

*RV(+), rotavirus antigen positive; RV(–), rotavirus antigen negative*.

* *Statistically significant*.

### Symptoms of RV Infection

**Figure 2:**
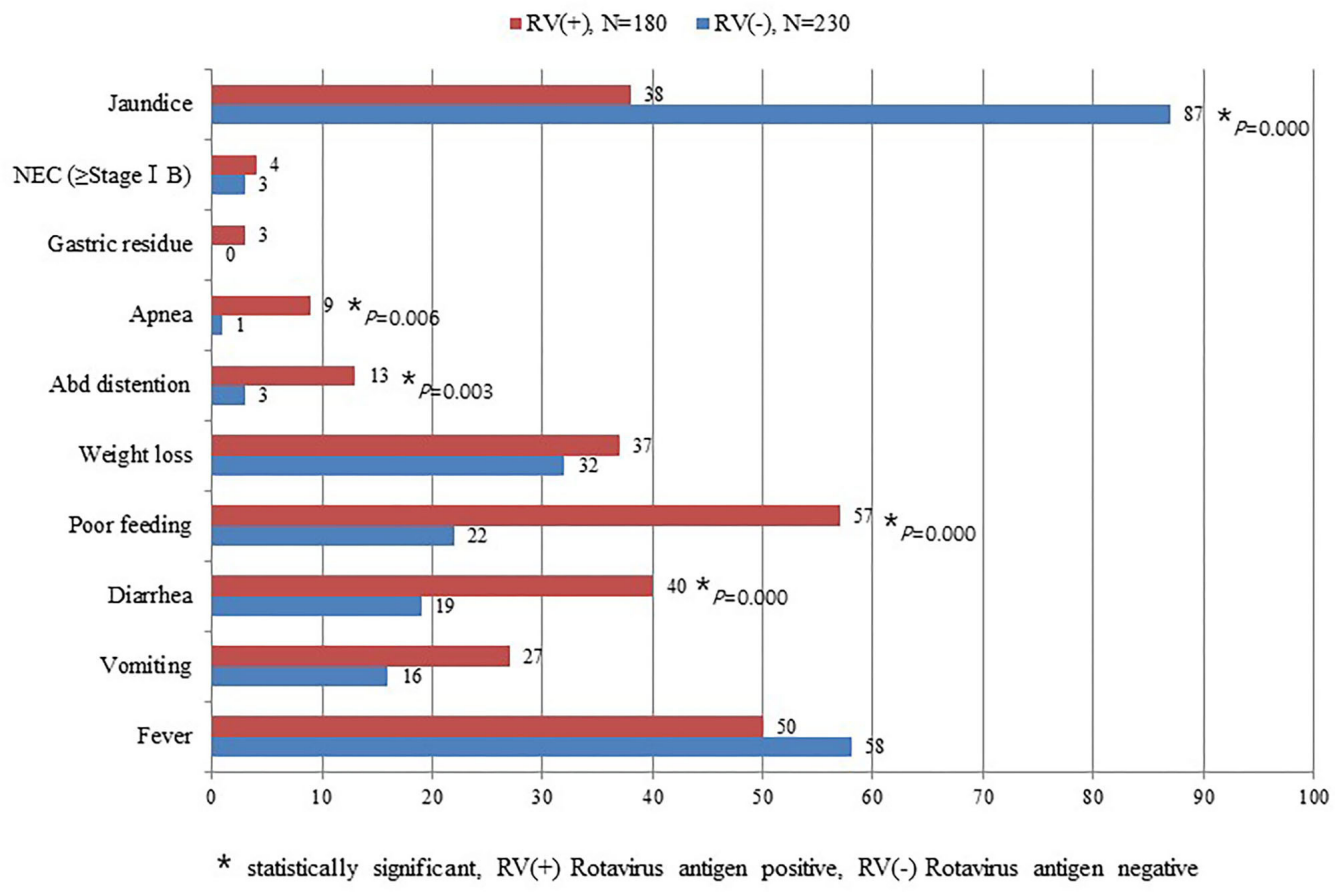
Comparison of clinical symptoms between patients with and without rotavirus infection.

**Figure 3:**
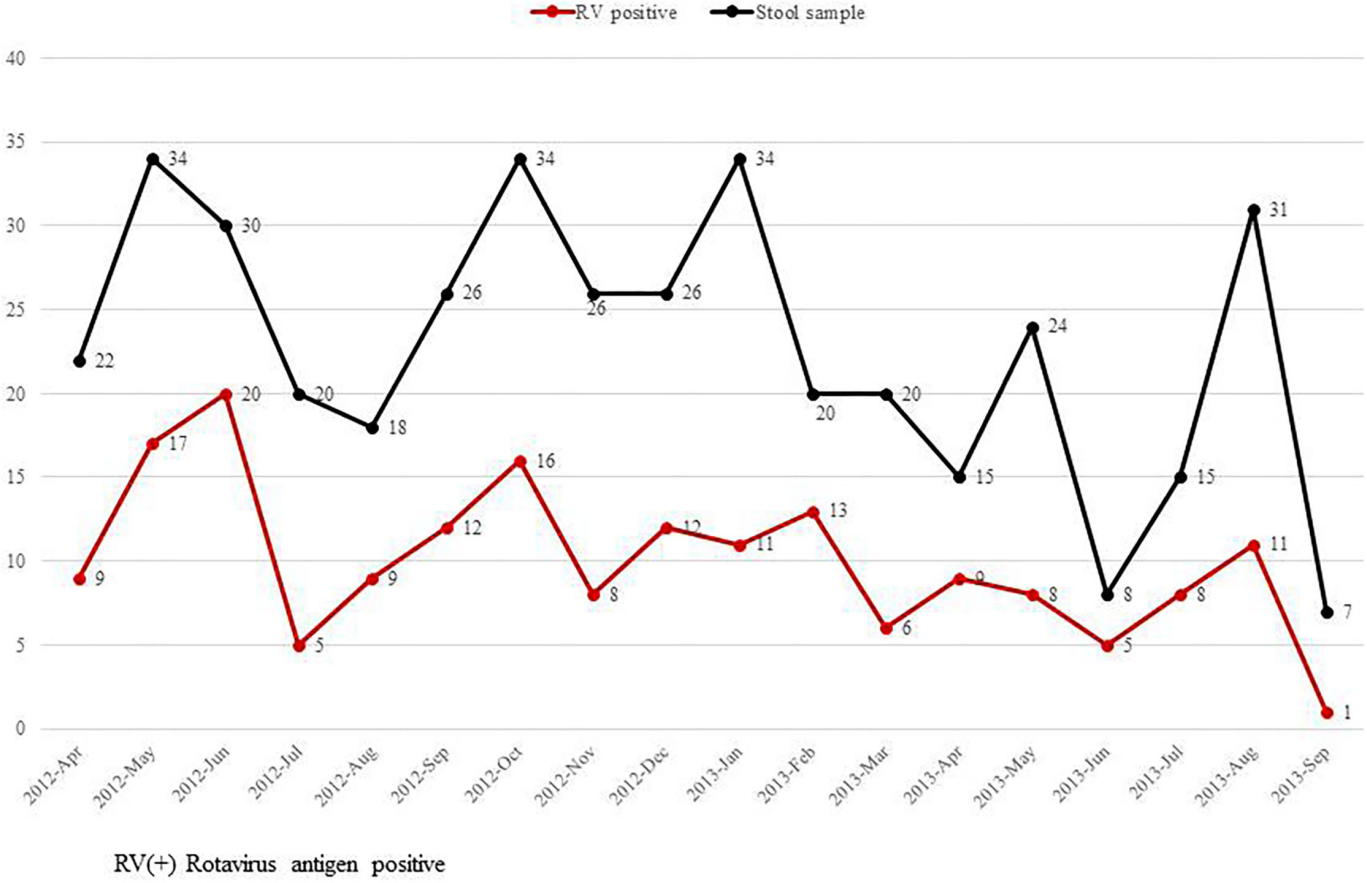
Monthly incidence of rotavirus infection in the neonatal intensive care unit from April 20, 2012 to September 10, 2013.

Symptoms of diarrhea, poor feeding, abdominal distention, and apnea were significantly higher among infants with RV infection than those without RV infection. In contrast, jaundice was significantly higher in the group without RV infection than those with RV infection (Figure 2). This study did not detect seasonal variation of RV infection in the NICU (Figure 3).

### RV Infection According to the Place of Care

**Figure 4:**
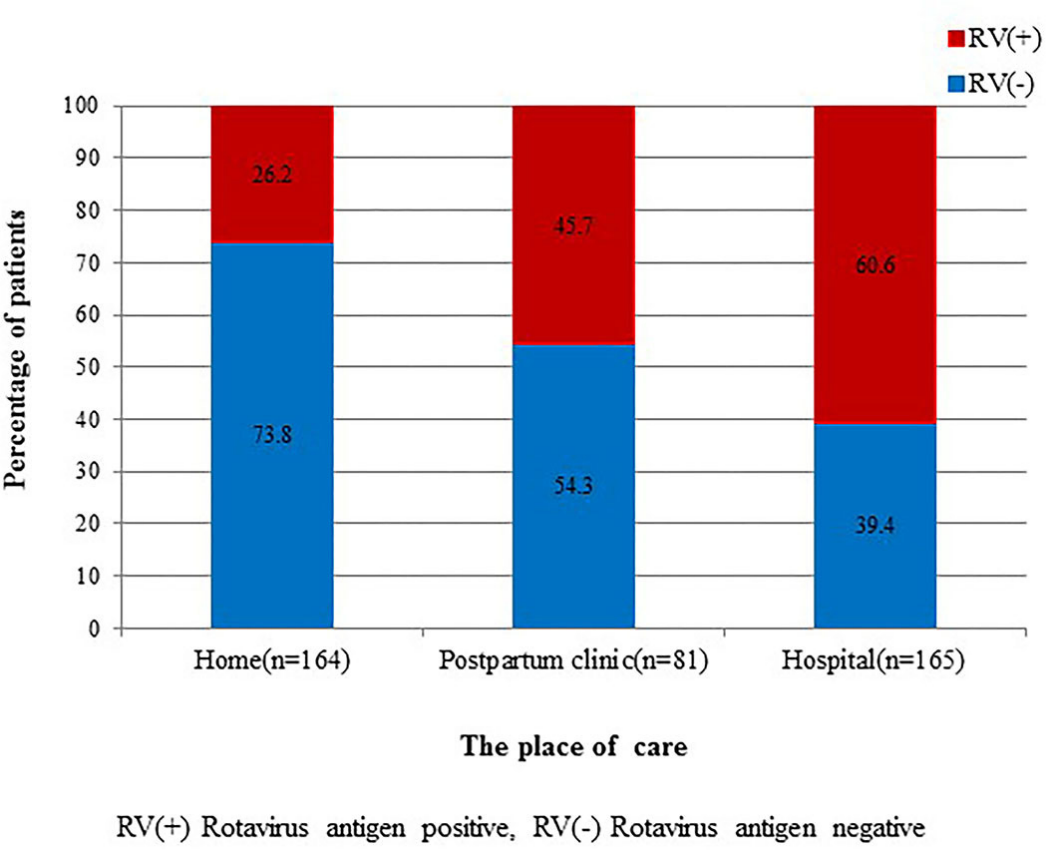
Comparison of rotavirus infection among admitted cases from home, postpartum clinic, and hospital. There are statistically significant differences among the three groups (*P* = 0.000).

We also examined where the infant was cared for within 48 h before admission; these places were divided into three groups: home, postpartum clinic, and hospital. We defined those cared for in a hospital as infants who were hospitalized after birth at Eulji University Hospital or infants who were born and stayed at other hospitals. Incidence of RV infection among the three groups, home (26.1%), postpartum clinic (45.1%), and hospital (60.2%), showed statistically significant differences (Figure 4).

### Genotype of RV

**Figure 5:**
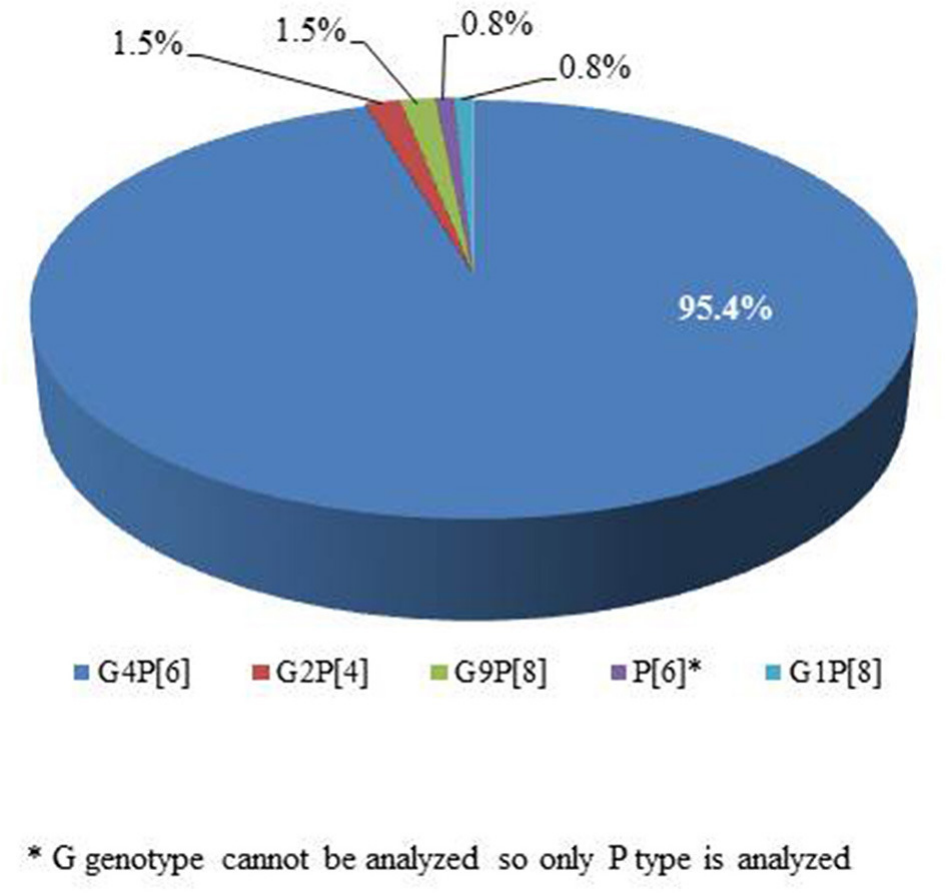
Genotype distribution of positive rotavirus cases in a neonatal intensive care unit (*n* = 131).

RV genotyping was performed on 180 patients who were positive for RV antigen. Two of them lacked specimens, and 47 did not proceed due to a lack of gene amplification or band interpretation (Figure 1). Genotypes were identified in 131 of the 180 patients with G4P[6] being predominant in 125 patients (95.4%; Figure 5). According to the genotype distribution by the place of care, the G4P[6] genotype was found 88.9, 96.3, and 97.4% in a home, postpartum clinic and a hospital, respectively. Genotypic differences among the three groups were not statistically significant (*P* = 0.237).

## Discussion

We investigated the incidence and symptoms of RV infection and genotype of RV in a NICU 5 years after the introduction of RV vaccines in South Korea. The overall incidence of RV infection in the NICU was 43.9%. The presence of RV antigen was highest among preterm infants, low birth weight infants, inborn infants, and infants born by cesarean section. Before the introduction of the RV vaccine, Shim et al. (19) reported that the incidence of RV infection in their NICU population was 25.2%, a population similar to ours. They also reported that infection rates were highest among preterm infants. Although the hospital and geographical region are not the same as in our study, this finding shows that the ineligible NICU population rarely develops herd immunity despite RV vaccination in the general population. Recently, Sahni et al. (20) reported that RV infection was higher in a NICU, which is a health care unit with low RV vaccine coverage. RV infection in newborn infants was known to be asymptomatic due to less pathogenic genotype infections and passive immunity transferred from mother to offspring (21). However, only 33 patients (18.3%) were asymptomatic and the remaining patients displayed symptoms in the current study. This result is similar to those of other studies in which the rate of asymptomatic infection ranged from 11 to 19% (14, 19). Moreover, RV infection is known as a risk factor for severe diseases, such as necrotizing enterocolitis (22) and periventricular leukomalacia in newborn and preterm infants (23, 24). Currently, around the world, there are different guidelines regarding RV vaccination for newborn and preterm infants in a NICU. However, RV vaccines are well-tolerated in NICU infants with no significant increase in nosocomial infection post-NICU-based vaccination and no difference in the risk of complications compared with the general population (25–27). Although we did not administer RV vaccination in our NICU population, our results suggest the need for RV vaccination according to the standard immunization schedule. Adhering to the routine schedule could reduce the incidence of RV infection in the ineligible NICU population, even if the concern of shedding due to vaccination remains.

Although studies on jaundice caused by bacterial infections, especially urinary tract infection, have progressed, studies on jaundice caused by viral infections are limited (28–30). Hwang and Kim (31) reported a relationship between asymptomatic RV infection and jaundice in newborns. However, in the current study, the association between RV-negative antigen and jaundice was statistically significant. Because the current study population included both asymptomatic and symptomatic RV infection groups, comparison of the two results is limited, and additional research is thus needed.

RV infection tends to be predominant in the winter (32). RV infections peaked in South Korea in winter and early spring (33–36). However, the current study did not detect seasonal variation possibly because of colonized RV transmission among the study groups. RV is known to be highly stable in the environment, and only a few virions are needed to cause infections in susceptible hosts (37). High transmission rates were suggested as possible factors facilitating the year-round circulation of RV (38). These findings indicate that RV can easily and quickly spread among newborn infants. Hence, it is important for the healthcare provider to implement basic precautionary measures, such as maintaining hygiene, isolation, and following good biosafety practices at work. Furthermore, the development of an RV vaccine for newborn infants is recommended. In the same context, we also found that the incidence of RV infection varies by place of infant care before hospital admission with the incidence of RV infection increasing in the order of home, postpartum clinic, and hospital. Group life of newborn like as hospital and postpartum clinic, which raised transmission rate of RV, affected to increase RV infection.

The dominant genotype in the NICU was G4P[6] among all groups in our study. This finding is consistent with the result reported by Mun et al. that G4P[6] was dominant in children under 1 year of age, including newborn infants (39). RV strains with the P[6] genotype are commonly detected in asymptomatic neonates in association with four major G-types (G1–G4) that colonize the NICU. However, P[6] strains have been reported as symptomatic genotypes in many countries, similar to our results (12, 40). Currently, studies on RV vaccine that can be suitable for the neonatal period are ongoing (11, 41, 42). There is a vaccine with the P[6] strain (11, 41), though current vaccines, such as Rotateq and Rotarix, do not contained the P[6] strain.

A limitation of this study is that it was conducted in a small group within a short period in a single medical institution. A total of 47 specimens could not be analyzed due to a lack of gene amplification or band interpretation. In addition, our study did not address infections with other viruses. Despite these limitations, this study is relevant in its evaluation of the effect of the introduction of RV vaccination in a NICU and measurement of RV genotype diversity in a NICU after the introduction of RV vaccines.

## Conclusions

Current RV vaccines in South Korea (RotaTeq and Rotarix) did not affect the incidence of RV in newborn and preterm infants in the NICU. Most patients with RV antigen positive results in the NICU had symptoms, and high incidence of RV infection was seen in hospital and postpartum clinic settings. The dominant RV genotype was G4P[6] across study groups.

## Data Availability Statement

The original contributions presented in the study are included in the article/supplementary material, further inquiries can be directed to the corresponding author/s.

## Ethics Statement

The study protocol was reviewed and approved by the Institutional Review Board of Eulji University Hospital (12-064). Written informed consent to participate in this study was provided by the participants' legal guardian/next of kin.

## Author Contributions

SK and HY: conception and design. SK, Y-HS, and HY: collection and assembly of data and drafting of the article. SK, Y-HS, and JL: analysis and interpretation of the data. SK, Y-HS, HY, and JL: critical revision of the article for important intellectual content and final approval of the article. All authors contributed to the article and approved the submitted version.

## Funding

This study was supported by an EMBRI Grant (2012-EMBRI-DJ0003) from the Eulji University.

## Conflict of Interest

The authors declare that the research was conducted in the absence of any commercial or financial relationships that could be construed as a potential conflict of interest.

## Publisher's Note

All claims expressed in this article are solely those of the authors and do not necessarily represent those of their affiliated organizations, or those of the publisher, the editors and the reviewers. Any product that may be evaluated in this article, or claim that may be made by its manufacturer, is not guaranteed or endorsed by the publisher.

## Abbreviations

RV: rotavirus
NICU: neonatal intensive care unit
PCR: polymerase chain reaction
RT-PCR: reverse transcription PCR.
